# Prevalence of *Mycoplasma genitalium* infection in women with bacterial vaginosis

**DOI:** 10.1186/s12905-020-00926-6

**Published:** 2020-03-26

**Authors:** Melinda B. Nye, Ayla B. Harris, Amanda J. Pherson, Charles P. Cartwright

**Affiliations:** 1 Center for Esoteric Testing, Laboratory Corporation of America® Holdings, Burlington, North Carolina, USA; 2 SpeeDx Pty. Ltd., Sydney, Australia

**Keywords:** Bacterial vaginosis, *Mycoplasma genitalium*, Prevalence, Association, Antimicrobial resistance

## Abstract

**Background:**

Bacterial vaginosis (BV) is a common condition in reproductive-age women and is known to be positively associated with risk of acquisition of sexually transmitted infections (STI) such as chlamydia and gonorrhea. *Mycoplasma genitalium* is an emerging STI that has been linked to increased risk of pelvic inflammatory disease, adverse pregnancy outcomes and infertility. In the present study we sought to examine whether women diagnosed with symptomatic BV were at increased risk of having concurrent infection with *Mycoplasma genitalium*.

**Methods:**

We used a novel PCR-based assay (*ResistancePlus* MG; SpeeDx Pty. Ltd., Sydney, Australia) to determine the prevalence of *Mycoplasma genitalium* infection and 23S rRNA macrolide-resistance mediating mutations (MRMM) in a cohort of 1532 women presenting with symptoms of vaginitis.

**Results:**

*M. genitalium* was detected in 4.0% (62/1532) of samples with 37.1% (23/62) harboring MRMMs. The prevalence of *M. genitalium* infection in subjects with BV was significantly higher than in subjects with non-BV vaginitis (7.0% v 3.6%; OR = 1.97 (95% CI: 1.14–3.39).

**Conclusions:**

Prevalence of *M. genitalium* infection is associated with BV in women with symptomatic vaginitis. Improved management of BV is needed as a component of STI prevention strategies.

## Background

Although *Mycoplasma genitalium* was first identified as a potential etiologic agent of non-gonococcal urethritis (NGU) in the early 1980’s [1], it was not until the advent of molecular amplification testing that the true significance of *M. genitalium* as a sexually transmitted infection (STI) could be accurately elucidated. Associations between *M. genitalium* and acute and chronic conditions in women, including cervicitis [2], pelvic inflammatory disease (PID) [3, 4] infertility [3, 4] and preterm birth [3, 4] have been established. In response to these findings, published guidelines for the management of STIs continue to be revised to reflect the need for assessment of *M. genitalium* in patients at risk of STI [5–7].

Bacterial vaginosis (BV) is the most prevalent cause of vaginitis symptoms in reproductive age women [8] and has been linked to increased risk of acquisition of other STIs, including Human Immunodeficiency Virus (HIV) [9], *Chlamydia trachomatis* [10] and *Neisseria gonorrhoeae* [10]. Testing for these STI agents in women diagnosed with symptomatic BV is recommended [7]. Only limited data is available on the prevalence and incidence of *M. genitalium* infection in women with BV, and more information is clearly needed. In a large study of asymptomatic, sexually active, university students conducted in the United Kingdom [11], overall *M. genitalium* prevalence was 3.3% (78/2378), this was, however, substantially elevated (6.5% v 2.4%) in women with BV. In a smaller US study of 400 female patients attending an urban STI clinic, an *M. genitalium* prevalence of 17.5% (70/400) was observed and no association between *M. genitalium* and BV was identified [12].

The present study adds to the relatively limited body of data [11–15] in which the prevalence of *M. genitalium* has been determined in women with BV. In this investigation we used a novel multiplexed PCR assay [16] (*ResistancePlus*® MG, SpeeDx Pty. Ltd., Sydney, Australia) to identify *M. genitalium* positive samples, and detect macrolide-resistance, in a large cohort of women with vaginitis [17].

## Methods

Residual vaginal swab samples were available for analysis from 1532 of the 1579 women originally enrolled in a previously published clinical validation study of a molecular assay for BV (NS-002) [17]. All subjects were aged between 18 and 50 and had presented at 1 of 5 locations in the US between August 2016 and March 2017 with symptoms of vaginitis. The sample series consisted of 2 vaginal swab samples collected in liquid Amies transport medium (Copan Diagnostics) that were used for Gram stain preparation and yeast culture, 1 vaginal swab sample collected in the Affirm™ VPIII transport system (Becton-Dickinson), and 2 APTIMA® vaginal swab collections (Hologic). One of the APTIMA® collections was used for performance of nucleic-acid amplification testing including assays for *M. genitalium*, BV, *Trichomonas vaginalis* and *Candida* spp.; the second APTIMA® was retained for microbiome analysis. Samples were analyzed for potential etiologies of vaginitis as follows:

### Bacterial vaginosis (BV)

Vaginal discharge was analyzed on each subject at enrollment according to Amsel criteria [18] with an 'Amsel positive’ sample having at least 3 positive results; a pH value of greater than 4.5, a positive “whiff test" (“fishy' odor upon addition of KOH), presence of clue cells upon microscopic evaluation, and thin, homogeneous vaginal discharge. Analysis by quantitative Gram-stain was performed at a central reference laboratory as described previously [19] and scored using the Nugent criteria [20]. Only samples that generated positive Nugent scores [7–10] and were positive by Amsel criteria were deemed to be BV positive.

### Vulvovaginal candidiasis (VVC)

The presence of *Candida* spp. in the samples was determined by use of 2 multiplexed PCR assays that enable detection and differentiation of *C. albicans* and *C. glabrata* (CAN-PCR) or *C. lusitaniae*, *C. krusei, C. parapsilosis* gp, and *C. tropicalis* (CAN2-PCR). Primer sequences for the CAN-PCR assay were disclosed in Cartwright et al. (2013) [21] and for CAN2-PCR are shown in Supplemental Table 1. Confirmation of the identity of Candida spp. detected in samples using PCR was performed by sequence analysis of the 18S rRNA-28S rRNA internally transcribed spacer (ITS) region.

### Trichomonas vaginitis (TV)

Trichomonas-containing samples were identified using the FDA-cleared APTIMA® *Trichomonas vaginalis* NAAT assay (Hologic Inc., San Diego, CA) with testing being performed according to the manufacturer’s instructions.

Testing for *M. genitalium* was performed using the ***Resistance****Plus*® MG (RPMG) assay. RPMG is a recently developed multiplexed PCR assay that enables simultaneous detection of *M. genitalium* DNA and a cluster of mutations in the 23S rRNA gene (A2058G, A2059G, A2058T, A2058C, A2059C) associated with macrolide resistance in this organism [16]. Previous studies have reported favorably on the performance of RPMG when compared with reference molecular methods for detection and resistance determination in *M. genitalium* [16]. Nucleic-acid was extracted from APTIMA vaginal swab collections (200 μL) using the MagNA Pure 96 System (Roche Molecular Diagnostics, Indianapolis, IN) as previously described with 200 μL input and 100 μl elution volumes. RPMG assays were performed in 20 μL reaction volumes using 5 μL of eluted nucleic acid using an ABI 7500 Fast Dx instrument (Thermo Fisher Scientific, Waltham, MA) with amplification parameters provided by the assay manufacturer. Three channels were used for product detection; one for *M. genitalium* detection based on amplification of the MgPa gene, a second for stacked detection of 23 s rRNA macrolide-resistance mediating mutations (MRMM), and the third for detection of an internal control target added prior to nucleic-acid extraction. Data reduction was performed using the FastFinder analysis software application (UgenTec NV, Hasselt, Belgium) provided by SpeeDx.

Statistical analyses were performed using the MedCalc® software suite (www.medcalc.org, Ostend, Belgium).

## Results

The overall prevalence of *M. genitalium* in the study cohort was 4.0% (62/1532) of which 37.1% (23/62) possessed MRMM. Univariate analyses of *M. genitalium* prevalence by microbiologic, clinical and demographic characteristics is shown in Table 1. *M. genitalium* (*p*-value = .020) infections were significantly more common in women with BV than those without this condition (OR = 1.97 (95% CI: 1.14–3.39); Table 1). *M. genitalium* prevalence was not, however, associated with the other etiologies of vaginitis (VVC and TV). In addition, independent analysis of the two diagnostic approaches for identification of BV showed a positive association with positive Nugent scores [7–10] but not with positive Amsel results (Table 1). The prevalence of *M. genitalium* in subjects self-identified as African-American was significantly higher than in non-African American subjects (6.4% v 2.9%, *p* = 0.002; OR: 2.33) and in subjects under age 25 versus those 25 and older (6.8% v 2.8%, *p* < 0.001; OR: 2.40). BV, African-American race, and age <  25 all remained independently associated with increased *M. genitalium* prevalence in multivariate analysis (Table 2).

**Table 1:** Characteristics of women enrolled in NS-002 study and association with prevalent *M. genitalium* infection

	***M. genitalium*** positive		
Characteristic	(***n*** = 62)	***p***-value	OR (95% CI)
**BV**			
Positive (307)	20 (7.0%)		
Negative (1225)	42 (3.6%)	0.020	2.12 (1.10–3.10)
**Amsel Criteria**			
Positive (464)	22 (5.0%)		
Negative (1068)	40 (3.7%)	0.252	1.37 (0.80–2.32)
**Nugent Score**			
Positive (469)	27 (5.8%)		
Negative (1063)	35 (3.3%)	0.042	1.84 (1.21–3.09)
**VVC**			
Positive (274)	13 (4.7%)		
Negative (1258)	49 (3.9%)	0.536	1.22 (0.66–2.28)
**TV**			
Positive (89)	5 (5.6%)		
Negative (1443)	57 (4.0%)	0.462	1.42 (0.56–3.64)
**Race/Ethnicity**			
African-American (516)	33 (6.4%)		
Non-African American (1016)	29 (2.9%)	0.002	2.25 (1.35–3.73)
**Age**			
< 25 years (471)	32 (6.8%)		
≥ 25 years (1061)	30 (2.8%)	< 0.001	2.40 (1.44–4.00)

Abbreviations: *BV* Bacterial vaginosis, *VVC* Vulvovaginal candidiasis, *TV* Trichomonas vaginitis, *OR* Odds Ratio, *CI* Confidence Interval

**Table 2:** Results of logistic regression analysis including characteristics identified as associated with prevalent *M. genitalium* infection by univariate analysis

Characteristic	***p***-value	AOR (95% CI)
BV (Positive v Negative)	0.031	1.81 (1.04–3.04)
Age (< 25 years v ≥ 25 years)	0.004	2.52 (1.50–4.22)
Race (AA vs non-AA)	0.016	1.94 (1.13–3.36)

Abbreviations: *AA* African-American, *AOR* Adjusted Odds Ratio, *CI* Confidence Interval

The presence of MRMMs was identified in a significant minority 37.1% (23/62) of the *M. genitalium* positive samples using the ResistancePlus MG PCR assay. The prevalence of MRMM-harboring *M. genitalium* was slightly higher in women without concurrent BV (41.4% vs 33.3%) but this was not statistically significant.

## Discussion

The prevalence of *M. genitalium* infection was significantly higher in women presenting with vaginitis caused by BV, similar results to those reported by Oakeshott and colleagues who were the first to report an association between *M. genitalium* and asymptomatic BV [11]. We identified an OR of *M. genitalium* in BV-positive vs BV-negative women of 1.97, a value of 2.73 was reported for this parameter in the Oakeshott et al. study [11]. We also demonstrated that this association is related to dysbiosis and not clinical manifestations of BV since positive Nugent Gram-stain scores alone were independently associated with *M. genitalium* prevalence (Table 1). Since the present study was conducted retrospectively it was not possible to determine the impact of BV on incidence of *M. genitalium* infection. A recently published prospective study, however, found no evidence that therapeutic intervention in women with asymptomatic BV impacted *M. genitalium* incidence during a 12-month follow-up period [15]. Whether this finding reflects a true lack of causality between vaginal dysbiosis and *M. genitalium* acquisition or is indicative of failure of therapeutic intervention to eliminate BV could not be determined [15]. It is conceivable that the presence of a vaginal microbiome consistent with BV merely serves as a non-specific marker for selecting populations at higher risk of infection with other STI including *M. genitalium* or, somewhat less likely, that persistence of a dysbiotic microbiome is more likely if patients are co-infected with *M. genitalium*. Additional studies will hopefully resolve these questions.

In the study of Sena et al. [15], black race, age < 21 years, and prior-pregnancy were identified as being significantly associated with prevalent *M. genitalium* infection in women with BV. Only limited demographic data was collected on subjects in the present study but we were able to assess associations between race, age and prevalence of *M. genitalium* infection. Similar associations between African-American race (OR = 2.33) and age (< 25 years old vs 25 years old or older; OR = 2.40) were identified in our study population as seen in that of Sena et al. [15]. The potentially confounding influence of race and age on the observed association between BV and *M. genitalium* prevalence was assessed using logistic regression analysis. Under multivariate analysis, however, BV remained significantly associated with prevalent *M. genitalium* infection as did African-American race and age <  25 years (Table 2). The lack of an association between clinical manifestations of BV, as assessed by Amsel scores, and *M. genitalium* prevalence (Table 1) was not unexpected since positive associations between BV and *M. genitalium* have previously been demonstrated in entirely asymptomatic populations [11, 15]. Limitations notwithstanding, the findings presented here and in the study of Sena et al. [15] suggest that the presence of BV represents a significant risk factor for acquisition of *M. genitalium* and logically, therefore, that women with untreated BV are at elevated risk of developing symptomatic *M. genitalium* infections and, or, transmitting this organism to their sexual partners. Given that testing for *M. genitalium* in asymptomatic populations is not currently recommended, improved diagnosis and management of BV may be a useful approach to mitigating these risks.

Importantly, by using a novel *M. genitalium* NAAT assay we were able to determine not merely the prevalence of *M. genitalium* in the study population but also the frequency with which MRMM were present in *M. genitalium* positive samples. MRMMs were identified in a significant minority (37.1%) of *M. genitalium* infections, a result somewhat higher than the 30.8% reported previously in a cohort of females with asymptomatic *M. genitalium* infection in the US [22]. Although azithromycin remains recommended first-line therapy for *M. genitalium* in the US [7], that recommendation pre-dates much of our understanding of the prevalence of macrolide-resistance in this organism and is likely to change in the relatively near future. For efforts at curtailing resistance to be successful, however, clearly consideration must be given to the importance of potential reservoirs of resistant organisms such as the vaginal milieu of asymptomatic women with dysbiotic microbiota.

## Conclusions

The present study extends previous findings regarding the epidemiology of *M. genitalium* infection, demonstrating that prevalence of infection with this organism is associated with BV in women with vaginitis. The results presented here demonstrate that improved diagnosis and management of vaginitis due to BV may prove to useful in decreasing prevalence and transmission of pathogenic organisms like *M. genitalium.*

## Supplementary information

**Additional file 1: Table S1.** Primer sequences used in the CAN2-PCR assay for *Candida krusei*, *Candida lusitaniae*, *Candida parapsilosis* gp. and *Candida tropicalis*.

## Data Availability

All data generated or analyzed during this study are included in this published article and supplementary information files.

## Abbreviations

AOR: Adjusted odds ratio
BV: Bacterial vaginosis
HIV: Human immunodeficiency virus
MRMM: Macrolide-resistance mediating mutations
NGU: Non-gonococcal urethritis
OR: Odds ratio
PID: Pelvic inflammatory disease
RPMG: ResistancePlus MG
STI: Sexually transmitted infection
TV: Trichomonas vaginitis
VVC: Vulvovaginal candidiasis
