# Supplementary material for: Prevalence of Mycoplasma genitalium infection in women with bacterial vaginosis
Source: BMC Womens Health. 2020 Mar 26;20:62. doi: 10.1186/s12905-020-00926-6 (PMC7099815; doi:10.1186/s12905-020-00926-6)
Supplement: Supplementary file 1 — Additional file 1: Table S1. Primer sequences used in the CAN2-PCR assay for Candida krusei, Candida lusitaniae, Candida parapsilosis gp. and Candida tropicalis. [file 12905_2020_926_MOESM1_ESM.docx]

**Supplemental Table 1**. Primer sequences used in the CAN2-PCR assay for *Candida krusei*, *Candida lusitaniae*, *Candida parapsilosis* gp. and *Candida tropicalis*.

| Target | Primer^1^ | Sequence (5’ – 3’) | 5’-location^2^ | GenBank no. |
| --- | --- | --- | --- | --- |
|  |  |  |  |  |
| *C. krusei* | CANFP-Ck | CGA.AGC.TGG.CCG.AGC.GAA.CT | 354 | KC408976 |
|  |  |  |  |  |
| *C. lusitaniae* | CANFP-Cl | GT.TGC.TCC.GAA.ATA.TCA.ACC.G | 244 | KC408980 |
|  |  |  |  |  |
| *C. parapsilosis* gp. | CANFP-Cp | TCC.ACT.CAT.TGG.TAC.AAA.CTC.C | 395 | KC408986 |
|  |  |  |  |  |
| *C. tropicalis* | CANFP-Ct | TGG.CCA.CCA.CAA.TTT.ATT.TCA.TAA.C | 409 | KC408968 |
|  |  |  |  |  |
|  |  |  |  |  |
| *Candida sp.* | CANRP-CS | FAM-isodC-CGT.AGG.ACA.GCG.GGT.AGT.CCT.ACC.TGA | 513^3^ | KU095863 |
|  |  |  |  |  |

^1^Species-specific forward primers located within the Internally Transcribed Spacer 2 (ITS2) region of the fungal rRNA operon.

^2^Indicates location of the 5’-terminal nucleotide position within the specified GenBank accession number.

^3^Pan-fungal reverse primer located within the 28S rRNA gene; FAM, 5-carboxyfluorescein, is used as the reporter dye coupled to the initial 5’-nucleotide in the primer; iso-dC, 2’-deoxy-5-methyl-isocytidine, is the initial nucleotide at the 5’ end of the fluorescently labeled primer.
